# Glucose control and cardiovascular outcomes: reorienting approach

**DOI:** 10.3389/fendo.2012.00110

**Published:** 2012-08-29

**Authors:** Romesh Khardori, Diep D. Nguyen

**Affiliations:** Division of Endocrinology and Metabolism, The EVMS Strelitz Center for Diabetes and Endocrine Disorders, Department of Internal Medicine, Eastern Virginia Medical SchoolNorfolk, VA, USA

**Keywords:** diabetes, treatment, insulin, heart disease, tight control

## Abstract

Cardiovascular disease accounts for nearly 70% of morbidity and mortality in patients with diabetes mellitus. Strides made in diabetes care have indeed helped prevent or reduce the burden of microvascular complications in both type 1 and type 2 diabetes. However, the same cannot be said about macrovascular disease in diabetes. Several prospective trials so far have failed to provide conclusive evidence of the superiority of glycemic control in reducing macrovascular complications or death rates in people with advanced disease or those with long duration of diabetes. There are trends that suggest that benefits are restricted to those with lesser burden and shorter duration of disease. Furthermore, it is also suggested that benefits might accrue but it would take a longer time to manifest. Clinicians are faced with the challenge to decide how to triage patients for intensified care vs less intense care. This review focuses on evidence and attempts to provide a balanced view of the literature that has radically affected how physicians treat patients with macrovascular disease. It also takes cognizance of the fact that the natural course of the disease may be changing as well, possibly related to better overall awareness and possibly improved access to information about better individual healthcare. The review further takes note of some hard held notions about the pathobiology of the disease that must be interpreted with caution in light of new and emerging data. In light of recent developments ADA and EASD have taken step to provide some guidance to clinicians through a joint position statement. A lot more research would be required to figure out how best to manage macrovascular disease in diabetes mellitus. Glucocentric stance would need to be reconsidered, and attention paid to concurrent multifactorial interventions that seem to be effective in reducing vascular outcomes.

## INTRODUCTION

The prevalence of diabetes is increasing globally mainly through the increase in the burden of type 2 diabetes. In the United States, prevalence of diabetes is estimated at 12.9% (Cowie et al., 2009). For those diagnosed during middle age, diabetes is associated with 10 years of lost life (Narayan et al., 2003). Much of diabetes related morbidity and mortality relate to cardiovascular disease (CVD; microvascular and macrovascular), and only through reduction in these vascular complications, would diabetic patients be able to achieve a quality (and quantity) of life similar to that enjoyed by their otherwise healthy counterparts. Patients with diabetes are at two- to fourfold greater risk for CVD (Kannel and McGee, 1979). This risk persists even after discounting smoking, hypertension, and dyslipidemia. These observations directly and indirectly implicate dysglycemia in the residual increased risk of vascular disease. This, however, should not detract from the cardiovascular (CV) benefits of blood pressure control and cholesterol lowering in patients with diabetes mellitus.

## HYPERGLYCEMIA AND CARDIOVASCULAR SYSTEM

A complex interaction exists that includes atherogenic dyslipidemia (a constellation of high triglycerides, low HDL, and increase in small dense LDL), chronic kidney disease, and autonomic dysfunction. A direct acceleration of atherogenesis has been attributed to insulin resistance and consequent hyperglycemia resulting in endothelial dysfunction, activation of platelets, activation of protein kinase-C, and formation of advanced glycation end products. Additionally, production of reactive oxygen species through activation of *NF-κB* is thought to be crucial to the development of vascular disease. Two recent reviews detail most of these mechanisms (Mazzone et al., 2008; Dandona et al., 2009). Whether steps elucidated in scores of papers dedicated to atherogenesis in diabetes indeed actually reflect true cause and effect relationship remain to be fully validated.

## EVIDENCE LINKING INCREASED GLUCOSE LEVEL WITH INCREASED CV-RISKS

Epidemiological evidence supports diabetes as a risk factor for CVD, and microvascular complications such as nephropathy and retinopathy. The relation between hyperglycemia and diabetes has been intensively studied (Coutinho et al., 1999; The Emerging Risk Factors Collaboration, 2010). Intensive glycemic control has been suggested to effectively reduce burden of micro and macrovascular disease in people with diabetes (Stratton et al., 2000; Adler et al., 2002).

While observational studies generally show a linear relationship between CVD and elevated glucose, there is possibly a breakpoint near or below the threshold for diabetes. The atherosclerosis risk in a community study showed a non-linear relationship to relative risk of coronary heart disease (CHD) and hemoglobin A_1C_ in non-diabetic adults (Selvin et al., 2005). An HbA_1C_ level below 4.6% was not related to CHD risk, but was significantly related to risk above that level.

In about 10,000 individuals without diagnosis of diabetes, the AusDiab study reported a “J-shaped” relations between CV mortality and fasting glucose besides a continuous increased risk for CV mortality with increasing HbA_1C_ and 2 h postprandial glucose during an oral glucose tolerance test (Barr et al., 2009). In an earlier study, Wei et al. (2000) had shown a “U” shaped relationship with low fasting plasma glucose as predictor of CVD and all-cause mortality.

The United Kingdom Prospective Diabetes Study (UKPDS) reported that the relationship between hyperglycemia and CV mortality is a continuum that starts at glucose level below the threshold for diagnosis of diabetes. It has been suggested that a 1% decrease in HbA_1C_ should be associated with a 14% decrease in relative risk for myocardial infarction (MI; Stratton et al., 2000).

Recently, however, analysis of participants in Ludwigshafen Risk and Cardiovascular Health Study of patients without history of diabetes undergoing coronary angiography showed a “J” shaped relationship between glycated hemoglobin, and cardiovascular and cancer mortality (Silbernagel et al., 2011). In a large cohort of elderly patients with diabetes (*n* = 28,000) generated from the UK General Practice Research Database, a “U” shaped association between HbA_1C_ levels and CV events has been suggested with lowest hazard ratio (HR) at an HbA_1C_ level of approximately 7.5% (Currie et al., 2010). This led to open advocacy for re-considering the one size fits all, approach in management of people with longstanding diabetes drawing customary fire from the organized specialty societies – coming as close as it did to the disturbing/surprising results from the Action to Control Cardiovascular Risk in Diabetes (ACCORD) trial (Mitka, 2010).

## GLYCEMIA CONTROL AND CV OUTCOMES

Relationship between glycemia and CV outcomes has been tested in well-designed interventional trials of intensified glycemic control. Four such studies deserve serious consideration (UKPDS follow-up, ACCORD, ADVANCE, and VADT; **Table 1**).

**Table 1:** Clinical characteristics and outcomes of intensive glucose lowering vs standard therapy on primary end point and mortality.

Characteristics	ACCORD	ADVANCE	VADT	UKPDS follow-up
*n*	10,251	11,140	1,791	3,277
Men/women (%)	61/39	58/42	97/3	61/39 (overall)
Mean age (year)	62	66	60	53
Mean duration of diastolic (year)	10	8	11.5	Newly diagnosed
Median HbA_1C_ at entry (%)	8.1	7.2	9.5	7.0
Median HbA_1C_ at study end (%)	6.4 vs 7.5*	6.4 vs 7.0*	6.9 vs 8.4*	7.0 vs 7.9*
Cardiovascular disease (%)	35	32	40	NA
Cardiovascular death (%)	↑ 35 (*P* = 0.02)	↓ 12 (*P* = NS)	↑ 25 (*P* = NS)	NA
Any death (%)	↑ 22 (*P* = 0.04)	↓ 7 (*P* = NS)	↑ 6.5 (*P* = NS)	NA
Severe hypoglycemia (%)	16.2 vs 5.1*	2.7 vs 1.0*	21.1 vs 9.9*	Variable**

* Standard study,

** variable depending on the type of medication.

The ACCORD Study included 10,251 patients with established type 2 diabetes, and one-third having had a cardiovascular event (The Action to Control Cardiovascular Risk in Diabetes Study Group, 2008). Patients were randomized to intensive glucose control (targeting an HbA_1C_ < 6.0% and achieving a level of 6.4%) or standard therapy (targeting HbA_1C_ of 7.0–7.9% and achieving level of 7.5%). A variety of glucose lowering therapies was used. There was a non-significant trend toward reduction in primary outcome of trial (a composite of non-fatal MI, non-fatal stroke, or death from CV causes) with intensive control. However, unexpectedly there was higher all-cause mortality (HR 1.22, 95% CI 1.01–1.46, *P* = 0.04) in the intensive control group. Higher rates of severe hypoglycemia for subjects in the intensified control group were reported. Patients with higher HbA_1C_ at baseline were at higher risk for hypoglycemia as were those who did not respond promptly with fall in HbA_1C_ in the intensified control group.

The ADVANCE Study was conducted to determine whether intensive lowering would reduce risk of microvascular and macrovascular events in individuals with type 2 diabetes and vascular risk factors-compared to standard conventional care (The ADVANCE Collaborative Group, 2008). The study involved 11,140 subjects. The mean duration of follow-up was 5 years. Mean HbA_1C_ achieved was 6.5% in the intensive therapy group compared with 7.3% in the standard group. Subjects in this intensive glycemic arm all received modified-release sulfonylurea (gliclazide) plus other glucose lowering therapies as needed to achieve glucose control. The CV component of the primary endpoint (a component of MI, stroke, and CV death) was not significantly reduced by intensified glucose control. The incidence of combined major and microvascular events was significantly reduced (HR 0.9, 95% CI 0.82–0.98, *P* = 0.01) in the intensive glucose control group. This was largely driven by reduction in progression of albuminuria or emergence of new nephropathy. There was no evidence of increase in CV or all-cause mortality in the intensified control group. Actually there was a non-significant trend toward reduction in all-cause mortality (HR 0.093, 95% CI 0.83–1.06).

The VADT Study included 1,791 American veterans, and 90% were males. A variety of glucose lowering agents was used including metformin, glimepiride, rosiglitazone, and insulin (Duckworth et al., 2009). An HbA_1C_ of 6.9% was achieved in intensified control arm compared with HbA_1C_ of 8.4% in standard treatment arm. After a median follow-up of 6.5 years, no significant lowering of composite CV outcomes was noted in the intensive control group. However, a borderline significant reduction in albuminuria was seen in this group. Benefits of intensive control were seen in those with shorter duration of diabetes, lower HbA_1C_, and absence of CVD at baseline. Coronary calcium scores predicted higher CV events in those with highest coronary calcium scores. A wide range of coronary calcium scores have been described in patients with diabetes. Severe hypoglycemia was more prevalent in the intensive control arm.

In the initial (18,19)” in the paragraph “In the initial UKPDS trial, 3,867 newly diagnosed subjects with type 2 diabetes were randomized to an intensive glucose control arm involving use of sulfonylureas or insulin, and a conventional arm employing lifestyle management. Over the 10-year period of trial those in the intensified control arm achieved a mean HbA_1C_ level of 7.0% compared with mean HbA_1C_ level of 7.9% in control arm. This degree of intensive control was associated with an approximately 1% decrease in HbA_1C_ and a non-significant 16% reduction in the risk of MI. There was significant reduction in the risk of microvascular complications (≈25%, 95% CI 7–14, *P* = 0.01). There was also a non-significant 6% reduction in all-cause mortality. However, there was no effect of intensive control or any other CVD outcome. A subgroup of overweight subjects was included in the study that compared intensive glucose control with metformin (*n* = 343) against conventional therapy described earlier (*n* = 411). Despite no significant difference in HbA_1C_, subjects treated with metformin showed a 39% relative risk reduction (RR) for MI (*P* = 0.001) and a 36% RR in all-cause mortality (UK Prospective Diabetes Study (UKPDS) Group, 1998; Holman et al., 2008).

The UKPDS follow-up study comprised all surviving subjects that completed the UKPDS randomized intervention trial in 1997. All subjects returned to usual physician care without any guidance from UKPDS investigators. Subjects were seen annually for collection of clinical and biochemical data between 1997 and 2001. Thereafter, between 2002 and 2007 information was gathered through mailed questionnaires. Over a third of patients who completed trial in 1997 were followed-up until 2007. Any difference between HbA_1C_ levels was lost in 1 year following completion of trial in 1997. In the postintervention follow-up period reduction in microvascular end points were maintained just like those seen during the intervention trial. The benefits of metformin therapy were also maintained. More interestingly and importantly, the glycemic control arm showed a significant 13% reduction in all-cause mortality and a 15% significant reduction in MI. In the metformin group RR persisted for any diabetes related end point (21%), MI (33%), and death from any cause (27%). These observations in patients with type 2 diabetes are similar to those seen in the Diabetes Control and Complications Trial (DCCT) follow-up – EDIC Study (EDIC Research Group, 1999) where difference in reduction of microvascular complications were maintained despite effacement of differences in HbA_1C_ levels. Furthermore, despite loss of glycemic separation, CV events, non-fatal MI, stroke, or CV deaths were reduced by 57% (The Diabetes Control and Complications Trial/Epidemiology of Diabetes Interventions and Complications (DCCT/EDIC) Study Research Group, DCCT/EDIC).

These persistent benefits generated from early strict glycemic control suggest a metabolic memory (also called “legacy effect”) that outlives original reduction in HbA_1C_ and subsequent loss of glycemic control. Epigenetic changes have been invoked as a mechanism to explain legacy effect.

It is worth mentioning that an earlier smaller study (Kumamoto Study) showed that glycemic control reduces CV events (50% lower in intensively treated subjects). The absolute number of events in this study was too low to draw any meaningful conclusions (Shichiri et al., 2000).

These positive influences of legacy effect need to be reconciled with unexpected deaths in the ACCORD trial. It seems that in patients with long standing diabetes and CVD, duration and magnitude of heart disease adversely affect the outcome if intense glucose control is forced where lowering of glycemic burden is difficult to accomplish as reflected in failure to affect prompt reduction in HbA_1C_. Thus caution is advised in patients with longer duration of diabetes and higher burden of CVD. On the other hand those with new onset or short duration of diabetes and no or lower burden of CVD should receive intensified glycemic control. This approach has been incorporated in the joint ADA–EASD position statement published in April 2012 (Inzucchi et al., 2012). This is a new direction moving away from hawkish stance of reducing glycated hemoglobin to less than 7% in all patients. It takes cognizance of the fact that intensified glucose control is fraught with increased risk (twofold increase) of severe hypoglycemia. Several meta-analysis have been carried out to sort the benefits and risks of tighter (intensive) glycemic control. These have been nicely discussed in a recent publication (Macisaac and Jerums, 2011). Recently two more meta-analysis have been published: the first one by Boussageon et al. (2011) found limited benefits of intensive glucose lowering on all-cause mortality and deaths from cardiovascular causes, while the second one by Hemmingsen et al. (2011) found that intensive glycemic control does not seem to reduce all-cause mortality in patients with type 2 diabetes, and that available data from randomized clinical trials remained insufficient to prove or refute relative RR for cardiovascular mortality, non-fatal MI, or composite microvascular complications. Furthermore, intensive glycemic control increased the relative risk of severe hypoglycemia by 30%.

## BOTTOM LINE

Interventional studies have been negative in the sense that they have failed to convincingly demonstrate the superiority of intensified glycemic control in reducing cardiovascular mortality. On the plus side, however, these studies have demonstrated that glucose lowering strategies are safe by and large, and may, over a period of time offer an advantage. The increased unexplained mortality seen in the ACCORD trial has dampened any further enthusiasm until these deaths are totally explained.

Undoubtedly, intensive therapies are associated with a greater risk of hypoglycemia, and that fear hovers over both the caregivers and the patients. Several interesting myths seem to have been shattered. (a) That intensified glucose control alone is sufficient to tilt the balance favorably in the short run is no longer a tenable proposition in sicker, older patients and those with long standing diabetes. (b) That insulin resistance is the major determinant of vascular disease in patients with diabetes deserves reconsideration. This is evident in many studies where rosiglitazone (an insulin sensitizer) was associated with more harm (Graham et al., 2010). Pioglitazone – another insulin sensitizer, fares no better when it comes to risks of acute MI in elderly patients.

Whether this is a consequence of facilitated insulin action following mitigation of insulin resistance remains to be proved (if so, it would actually suggest a cardio-protective role for insulin resistant state – a concept that is alien to current thinking). Furthermore it is important to consider whether modalities employed to reduce the glycemic load themselves might affect the outcome. This is particularly true when considering use of sulfonylureas that have been associated with adverse cardiac outcome (Riddle, 2010).

Insulin resistance as a precursor to evolution of clinical diabetes needs to be re-examined in light of unique personal omics profile just reported (Chen et al., 2012). In this fascinating longitudinal study tracing evolution of diabetes, insulin resistance did not precede onset of dysglycemia. (c) That intensive lowering of blood pressure in patients with diabetes should further improve cardiovascular outcomes also appears to be doubtful as seen in the ACCORD trial (The ACCORD Study Group, 2010a). (d) That targeting atherogenic dyslipidemia of diabetes using fibrates (↑TG, ↓HDL) may not yield any advantage over use of a statin alone (The ACCORD Study Group, 2010b).

New approaches are underway to find therapies better suited to favorably affect cardiovascular outcomes. In this regard studies pertaining to efficacy of “Incretins” and DPP-IV inhibitors as favorable modulators are being watched with great anticipation. How Incretins/DPP-IV inhibitors exert favorable effects remains to be fully sorted out. It is, however, reported that favorable effects may be linked to enhancement of left ventricular regional function, and enhancement of delivery of endothelial progenitor cells (EPCs) under influence of stromal cell-derived factor (SDF)-1 alpha which mobilizes the EPCs. SDF-1 alpha is a substrate for DPP-IV, and inhibition of DPP-IV will increase SDF-1 alpha concentration. A recent publication addresses cardiovascular effects of DPP-IV inhibitors in greater depth (Jose and Inzucchi, 2012).

## NEW THINKING

Evidence generally trumps intuition, and diabetes care is no exception. Treatment plans have to be developed to fit the needs and expectations of the index patient. Sweeping generalizations can no longer dictate the type and intensity of care. New data that have emerged in last 5 years should reorient the direction and thrust of research in diabetes.

## FINALLY THE GOOD NEWS

Death rates among both U.S. men and women with diabetes have declined substantially between 1997 and 2006. This has reduced the absolute difference between adults with and without diabetes. The rate of improvement among those with diabetes has exceeded improvement in those without diabetes. Still the excess mortality risks remain high, but these are significantly lower than before. Contrasting with observations from before, improvements are being noted in both men and women with diabetes (Gregg et al., 2012).

## SUMMARY

Cardiovascular disease in patients with diabetes mellitus remains one of the leading causes of morbidity and mortality. Attempts focusing on intensive glucose control to affect the outcomes remain disappointing thus far. In light of this prudent care would require cautious multifactorial intervention (Gaede et al., 2008). Clinicians must use evidence to support implementation of protocols often used to treat patients with diabetes mellitus outside of emergencies such as diabetic ketoacidosis and non-ketotic hyperglycemic-hyperosmolar states. Current evidence supports individualizing care rather than following a format that does not allow for individual variation.

## Conflict of Interest Statement

The authors declare that the research was conducted in the absence of any commercial or financial relationships that could be construed as a potential conflict of interest.
